# Prevention of Sarcopenia and Maintenance of Exercise Tolerance by Individualized Prehabilitation in a Patient With Esophageal Cancer During Preoperative Adjuvant Therapy: A Case Report

**DOI:** 10.7759/cureus.64633

**Published:** 2024-07-16

**Authors:** Ririko Sakamoto, Kazuki Okura, Yushi Nagaki, Akiyuki Wakita, Yusuke Sato

**Affiliations:** 1 Department of Rehabilitation Medicine, Akita University Hospital, Akita, JPN; 2 Department of Esophageal Surgery, Akita University Hospital, Akita, JPN

**Keywords:** preoperative adjuvant therapy, chemoradiotherapy, sarcopenia, prehabilitation, esophageal cancer

## Abstract

Preoperative adjuvant therapy for esophageal cancer increases sarcopenia and decreases exercise tolerance, which are risk factors for postoperative pneumonia. Preoperative rehabilitation for patients undergoing esophagectomy effectively reduces the incidence of postoperative pneumonia. Therefore, the risk factors should be optimized by preoperative rehabilitation. Our patient had several risk factors for postoperative pneumonia, including low exercise tolerance, presarcopenia, and low respiratory muscle strength. However, because of the patient's advanced age, multiple comorbidities, and poor nutritional status, we struggled to determine the appropriate exercise intensity. Furthermore, there was a concern that chemotherapy-related adverse events could prevent adequate exercise from being performed. However, with individualized measures such as adjustable exercise intensity settings based on treatment status and nutritional management through multidisciplinary collaboration, it was possible to prevent sarcopenia and maintain exercise tolerance during preoperative adjuvant therapy. Individualized support in preoperative rehabilitation was suggested to contribute to the prevention of sarcopenia and maintenance of exercise tolerance during preoperative adjuvant therapy.

## Introduction

Preoperative adjuvant therapy followed by surgical treatment is indicated for resectable locally advanced esophageal cancer [1]. Postoperative respiratory complications (PPC) are common due to the invasive nature of esophagectomy [2]. Prevention of PPC is crucial because it affects postoperative outcomes [3].

Numerous preoperative factors influence the development of PPC during thoracic surgery. Sarcopenia, exercise tolerance, inspiratory muscle weakness, and skeletal muscle loss during preoperative chemotherapy can be modified by rehabilitation [4-7]. Halliday et al. reported an association between preoperative rehabilitation and fewer postoperative cases of pneumonia in patients with esophageal cancer undergoing preoperative chemotherapy and recommended preoperative rehabilitation [8].

However, decreased exercise tolerance and increased sarcopenia due to preoperative adjuvant therapy have also been reported [9,10]. Therefore, patients receiving preoperative adjuvant therapy require individualized support according to their general conditions. Hence, clinicians are likely to devise practical measures.

In this case, the key issues for physical therapy were to maintain exercise tolerance, muscle strength, and skeletal muscle mass loss during preoperative adjuvant therapy. However, the patient had numerous inhibitory factors for exercise therapy, which made it difficult to perform sufficient exercise. We provided several individualized supports to accomplish the task, and the patient had a good postoperative outcome.

## Case presentation

The patient was a 72-year-old male (height: 169.5 cm, weight: 55.7 kg, BMI: 19.4 kg/m^2^). He was diagnosed with esophageal cancer in the middle thoracic region (cT3brN2M1b, cStage: IVB). He was admitted to our hospital for preoperative radiochemotherapy and surgical treatment. Comorbidities included chronic heart failure, paroxysmal atrial fibrillation, and chronic renal failure, with an ASA-PS score of 2. The patient was taking diltiazem hydrochloride, valsartan, febuxostat, and acetaminophen. Echocardiography showed left ventricular ejection fraction (62.5%), E/A (0.8), and deceleration time (177.0 ms). The patient had been consuming food orally since the first day of hospitalization (day 1) but was instructed to refrain from eating and drinking on day 7 because of esophageal dilatation observed on esophagography. Physical therapy was administered on day 7. The baseline assessment results are presented below. Grip strength was 35.0 kgf (dominant hand), maximum inspiratory pressure (MIP) was 16.0 cmH_^2^_O (%MIP: 23.6%), comfortable walking speed was 0.92 s/m, Short Physical Performance Battery (SPPB) score was 12 points, six-minute walking distance was 435.0 m, and Appendicular Skeletal Muscle Mass Index (ASMI) was 6.9 kg/m^2^ (Table 1). Appendicular skeletal muscle mass was measured using a bioelectrical impedance body composition analyzer (InBody770; InBody Japan, Tokyo, Japan). Nutritional status indicated a moderate nutritional risk with a Geriatric Nutritional Risk Index (GNRI) of 87.4. Nutritional status was assessed weekly in consultation with a dietitian. Radiotherapy and intravenous nutrition were initiated on day 9 [11]. The protein intake at this point was 0.7 g/kg/day. After consulting a nutritionist, we suggested that the physicians increase the patient's protein intake. The physicians were concerned about the worsening renal function due to chemotherapy and decided to increase the protein dosage after chemotherapy. We developed a physical therapy program consisting of resistance training, aerobic exercises, and inspiratory muscle training for 40 minutes/day, five days a week. All exercise intensities were set at the Borg Scale 13. Resistance training consisted of two sets of 20 repetitions each for shoulder and elbow flexion, squats, cuff raises, and hip extension. Aerobic exercise was performed using a stationary ergometer, gradually increasing the load to 30 watts for 15 min. Inspiratory muscle training (IMT) was performed using a digital inspiratory muscle trainer (POWER breathe KH2^®^; POWER breathe International Ltd., Southam, Warwickshire, UK) software. The training was performed twice daily for 30 breaths, and the training load was set at 30-40% of the MIP.

**Table 1:** Changes in risk factors for postoperative pneumonia. ASMI: Appendicular Skeletal Muscle Mass Index; SPPB: Short Physical Performance Battery; 5STS: five-time sit-to-stand test; MIP: maximal inspiratory pressure; 6MWD: six-minute walking distance

Variable	Day 7	Day 37	Day 53	Day 88	Cutoff value
ASMI (kg/m²)	6.9	6.3	6.3	6.4	7.0
Handgrip strength (kgf)	35.3	35.5	34.0	29.6	28
SPPB	12	12	12	12	9
5STS (s)	10.9	9.1	10.5	8.9	12
10m gait speed (m/s)	0.92	0.91	0.91	0.92	1.00
MIP (cmH_2_O)	16.0	21.0	26.0	31.7	60.0
%Predicted	23.6	39.6	38.3	46.7	80.0
6MWD (m)	435.0	440.0	440.0	510.0	454.0

**Figure 1:**
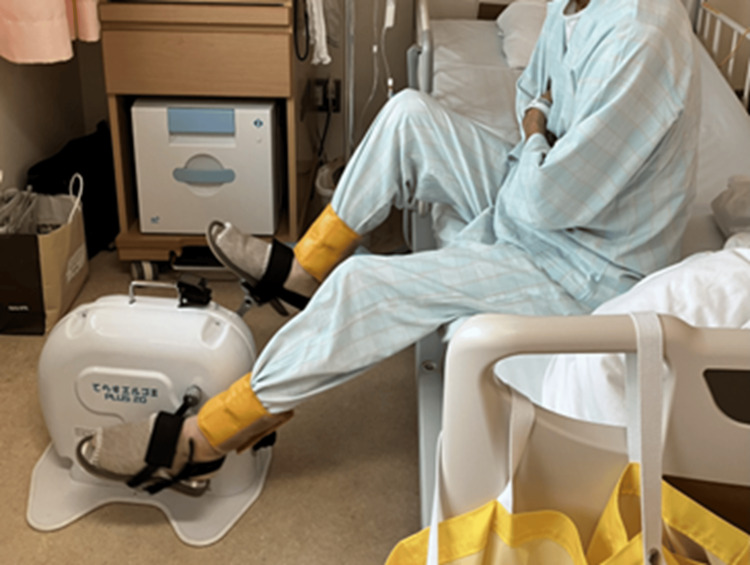
Picture during aerobic exercise. Aerobic exercise was performed at 20 W for 10 minutes in the hospital room using a portable compact ergometer (TERASU ERUGO®; Showa Denki Co., Ltd., Osaka, Japan).

80 mg/m^2^ nedaplatin was administered on day 14, and 400 mg/m^2^ 5-fluorouracil was administered from days 14 to 18 [11]. The protein dosage was increased to 1.1 g/kg/day on day 19. The patient experienced CTCAE Grade 1 nausea as a side effect of chemotherapy on days 16-25, Grade 1 fatigue on days 24-45, and increased pessimism during physical therapy. Therefore, the exercise intensity was reduced to a Borg Scale score of 11 during this period. Resistance exercises were performed as one set of 10 repetitions each, IMT was performed as one set of 30 repetitions at 30% MIP, and aerobic exercise was performed at 20 W for 10 min. Because of CTCAE Grade 2 leukopenia on days 31-41, physical therapy sessions were conducted in the hospital room to ensure infection control. Aerobic exercise was performed in the hospital room using a portable compact ergometer (TERASU ERUGO^®^; Showa Denki Co., Ltd., Osaka, Japan) at an intensity similar to that of a stationary ergometer (Figure 1).

On day 42, the white blood cell recovered to 3,500/mcL, and physical therapy was switched to the rehabilitation room the following day. Subjective exercise intensity was gradually increased to the Borg Scale scores of 11-13; eventually, the patient could exercise at the same intensity as at the beginning. On day 38, CT images from the intermediate evaluation confirmed the reduction in the main lesion and 112 aoP and the absence of non-ablative factors, and a final decision was made to proceed with surgery. Radiotherapy (41.4 Gy) was administered on day 42. Oral intake was initiated on day 43, and the patient was able to consume the entire gruel. The physical therapy evaluation on the day before discharge on day 53 is as follows: Grip strength was 34.5 kgf (dominant hand), MIP was 26.0 cmH_2_O (%MIP: 38.3%), comfortable walking speed was 0.92 sec/m, SPPB was 12 points, six-minute walking distance was 440.0 m, and ASMI was 6.3 kg/m^2^ (Table 1). The GNRI was 82.3. The patient was discharged on day 54. Because he had a low tolerance for IMT, we gave him a pamphlet and instructed him to continue resistance exercises during his home care.

## Discussion

This case suggests that individualized support helps maintain exercise tolerance and strength and prevents the loss of skeletal muscle mass in preoperative rehabilitation during adjuvant therapy. The patient had multiple risk factors for PPC at the start of rehabilitation. A preoperative six-minute walking distance of less than 454 m has been proposed as a cutoff value for postoperative complications in studies involving patients with esophageal cancer [12]. In this case, the baseline six-minute walking distance was 435.0 m, which was lower than the cutoff value. In terms of inspiratory muscle strength, a decrease of less than 80% of the predicted value is defined as a risk factor for PPC in patients with esophageal cancer. The patient had a MIP of 23.6% and low inspiratory muscle strength. Although the patient's baseline physical function was maintained, his appendicular skeletal muscle mass was below the sarcopenia criteria [13]. Based on these findings, the patient was considered to be at high risk of developing PPC. In contrast, decreased exercise tolerance and increased sarcopenia have been reported during preoperative adjuvant therapy [9,10]. Therefore, the key issue for physical therapy was to maintain exercise tolerance, muscle strength, and skeletal muscle mass during adjuvant therapy.

A skeletal muscle wasting rate of 4.44% was reported on a meta-analysis of skeletal muscle wasting during preoperative adjuvant therapy for esophageal and esophagogastric junction cancers [14]. In this case, the appendicular skeletal muscle wasting rate was 8.7%, which was significantly higher than that reported in previous studies. Preoperative radiochemotherapy, advanced age, and male sex were the risk factors for severe skeletal muscle loss during preoperative adjuvant therapy. The present case had all these factors, which may have predisposed the patients to the loss of skeletal muscle mass. On the other hand, a significantly higher prevalence of sarcopenia and postoperative pneumonia after chemotherapy in the severe skeletal muscle loss group is reported, defined as a skeletal muscle index change of 12% or more [7]. In the present case, the skeletal muscle mass was reduced but could be controlled to less than 12%. Guinan et al. reported that the handgrip strength of patients with esophageal cancer had a significant decrease of 4.3 kg during preoperative adjuvant therapy [10]. The reduction in this case was 1.3 kg, which is smaller than that in previous studies. The final grip strength was 34.5 kg, which is above the sarcopenia threshold of 28 kg [13]. Regarding exercise tolerance, preoperative adjuvant chemotherapy has been reported to be toxic and to cause a decrease in cardiopulmonary function. Therefore, exercise tolerance was expected to decrease without exercise therapy; however, the patient's tolerance to exercise was maintained [15]. Although the patient had a preoperative risk, he did not develop PPC and showed good progress. This was attributed to several modifications in preoperative rehabilitation that helped maintain exercise tolerance and muscle strength and prevented skeletal muscle mass loss.

In this study, we made three main modifications to the exercise prescription to address these problems. First, multidisciplinary nutritional management was combined with rehabilitation. Multidisciplinary rehabilitation has been reported to maintain skeletal muscle and grip strength during preoperative adjuvant chemotherapy [16]. The patient's nutritional status was poor, and there were concerns that the patient might not benefit from exercise therapy. With regard to nutritional management in cancer patients, ESPEN guidelines recommend an amino acid intake of 1.0-1.5 g/kg/day to prevent the loss of skeletal muscle mass [17]. During the physical therapy period, the patient consumed at least 1.0 g/kg/day of amino acids. Therefore, rehabilitation following nutritional management in collaboration with multiple professionals contributed to suppressing declines in handgrip strength and skeletal muscle mass. Second, the exercise intensity was flexibly set according to the treatment status. This was considered useful for maintaining exercise tolerance during preoperative adjuvant therapy. Cancer rehabilitation monitoring should focus on patient's self-reports of exercise tolerance [18]. Reports on healthy adults have demonstrated that eight weeks of training at a subjective exercise intensity of 13 improves exercise tolerance, according to the Borg Scale. Therefore, the exercise intensity in this case was also set at Borg Scale 13 [19]. Preoperative chemotherapy adversely affects patient adherence [17]. In the present case, there was concern that the patient had difficulty adhering to the exercise regimens because of the adverse effects of chemotherapy. Therefore, the intensity was reduced to Borg Scale 11, which is the maximum exercise intensity that the patient could tolerate.

As a result, the patient could continue exercise therapy despite many pessimistic comments during the period when the side effects occurred. Therefore, the flexible change in intensity according to the treatment status contributed to avoiding a decrease in patient adherence and maintaining exercise tolerance. Third, a portable compact ergometer was used for aerobic exercise during isolation for leukopenia. Reviews on cancer rehabilitation recommend that moderate exercise or light resistance exercise is acceptable when platelets are greater than 30.0×106/L and a symptomatic approach is recommended when leukocytes are less than 4.0×109/L or red blood cells are less than 11 g/dL [17]. In this case, myelosuppression reduced the platelets to 115.0×106/L, white blood cell count to 2.0×109/L, and red blood cells to 10.5 g/dL. Therefore, moderate-intensity exercise intensity was deemed appropriate based on the patient's symptoms. Moderate exercise intensity corresponds to a subjective exercise intensity of 12-13. Therefore, the patient was advised to continue the exercise therapy at a subjective intensity of 13 during myelosuppression. However, exercise therapy was limited to the hospital ward to prevent infection due to the low white blood cell count. Unlike when using a bicycle ergometer, it is difficult to quantify the exercise intensity while walking in the ward. Therefore, the patient could continue the aerobic exercise at a subjective intensity of 13 using a portable compact ergometer. This was useful in maintaining exercise intensity during myelosuppression and facilitating a smooth transition to aerobic exercise on a bicycle ergometer after leukocyte recovery.

## Conclusions

We managed the patient's nutritional needs with a multidisciplinary team, set the exercise intensity flexibly according to the treatment status, and selected exercise equipment appropriate for the environment. These individualized measures contributed to maintenance exercise tolerance, muscle strength, and skeletal muscle mass loss during preoperative adjuvant therapy, which may have led to the prevention of PPC. Preoperative adjuvant therapy has become the standard care for patients with esophageal cancer, and rehabilitation during this period is becoming increasingly important. Furthermore, patients with esophageal cancer are aging, and it is anticipated that the number of patients with multiple disincentives to exercise therapy, as in this case, will increase in the future. This report may assist in providing rehabilitation during preoperative adjuvant treatment for patients requiring individualized care.

## References

[1] Kitagawa Y, Ishihara R, Ishikawa H (2023). Esophageal cancer practice guidelines 2022 edited by the Japan esophageal society: part 1. Esophagus.

[2] Yang CK, Teng A, Lee DY, Rose K (2015). Pulmonary complications after major abdominal surgery: National Surgical Quality Improvement Program analysis. J Surg Res.

[3] Kinugasa S, Tachibana M, Yoshimura H (2004). Postoperative pulmonary complications are associated with worse short- and long-term outcomes after extended esophagectomy. J Surg Oncol.

[4] Fukushima T, Watanabe N, Okita Y (2023). The evaluation of the association between preoperative sarcopenia and postoperative pneumonia and factors for preoperative sarcopenia in patients undergoing thoracoscopic-laparoscopic esophagectomy for esophageal cancer. Surg Today.

[5] Hattori K, Matsuda T, Takagi Y (2018). Preoperative six-minute walk distance is associated with pneumonia after lung resection. Interact Cardiovasc Thorac Surg.

[6] Okura K, Suto A, Sato Y (2023). Preoperative inspiratory muscle weakness as a risk factor of postoperative pulmonary complications in patients with esophageal cancer. J Surg Oncol.

[7] Harada T, Tatematsu N, Ueno J (2022). Prognostic impact of postoperative loss of skeletal muscle mass in patients aged 70 years or older with esophageal cancer. Ann Surg Oncol.

[8] Halliday LJ, Doganay E, Wynter-Blyth VA, Hanna GB, Moorthy K (2021). The impact of prehabilitation on post-operative outcomes in oesophageal cancer surgery: a propensity score matched comparison. J Gastrointest Surg.

[9] Jack S, West MA, Raw D (2014). The effect of neoadjuvant chemotherapy on physical fitness and survival in patients undergoing oesophagogastric cancer surgery. Eur J Surg Oncol.

[10] Guinan EM, Doyle SL, Bennett AE (2018). Sarcopenia during neoadjuvant therapy for oesophageal cancer: characterising the impact on muscle strength and physical performance. Support Care Cancer.

[11] Wakita A, Motoyama S, Sato Y (2023). Preoperative neoadjuvant chemoradiotherapy provides borderline resectable thoracic esophageal cancer with equivalent treatment results as clinically T3 thoracic esophageal cancer. Ann Gastroenterol Surg.

[12] Inoue T, Ito S, Kanda M (2020). Preoperative six-minute walk distance as a predictor of postoperative complication in patients with esophageal cancer. Dis Esophagus.

[13] Chen LK, Woo J, Assantachai P (2020). Asian Working Group for Sarcopenia: 2019 consensus update on sarcopenia diagnosis and treatment. J Am Med Dir Assoc.

[14] Wang P, Wang S, Li X (2022). Skeletal muscle wasting during neoadjuvant therapy as a prognosticator in patients with esophageal and esophagogastric junction cancer: a systematic review and meta-analysis. Int J Surg.

[15] Halliday LJ, Boshier PR, Doganay E, Wynter-Blyth V, Buckley JP, Moorthy K (2023). The effects of prehabilitation on body composition in patients undergoing multimodal therapy for esophageal cancer. Dis Esophagus.

[16] Prado CM, Purcell SA, Laviano A (2020). Nutrition interventions to treat low muscle mass in cancer. J Cachexia Sarcopenia Muscle.

[17] Maltser S, Cristian A, Silver JK, Morris GS, Stout NL (2017). A focused review of safety considerations in cancer rehabilitation. PM R.

[18] Parfitt G, Evans H, Eston R (2012). Perceptually regulated training at RPE13 is pleasant and improves physical health. Med Sci Sports Exerc.

[19] Halliday LJ, Doganay E, Wynter-Blyth V, Osborn H, Buckley J, Moorthy K (2021). Adherence to pre-operative exercise and the response to prehabilitation in oesophageal cancer patients. J Gastrointest Surg.

